# Fabrication of All-SiC Fiber-Optic Pressure Sensors for High-Temperature Applications

**DOI:** 10.3390/s16101660

**Published:** 2016-10-17

**Authors:** Yonggang Jiang, Jian Li, Zhiwen Zhou, Xinggang Jiang, Deyuan Zhang

**Affiliations:** 1 School of Mechanical Engineering and Automation, Beihang University, Beijing 100191, China; jiangyg@buaa.edu.cn (Y.J.); lj379@outlook.com (J.L.); zzw89_177@126.com (Z.Z.); sdjxg@163.com (X.J.); 2 International Research Institute for Multidisciplinary Science, Beihang University, Beijing 100191, China

**Keywords:** pressure sensor, silicon carbide, ultrasonic assisted machining, bonding, harsh environment

## Abstract

Single-crystal silicon carbide (SiC)-based pressure sensors can be used in harsh environments, as they exhibit stable mechanical and electrical properties at elevated temperatures. A fiber-optic pressure sensor with an all-SiC sensor head was fabricated and is herein proposed. SiC sensor diaphragms were fabricated via an ultrasonic vibration mill-grinding (UVMG) method, which resulted in a small grinding force and low surface roughness. The sensor head was formed by hermetically bonding two layers of SiC using a nickel diffusion bonding method. The pressure sensor illustrated a good linearity in the range of 0.1–0.9 MPa, with a resolution of 0.27% F.S. (full scale) at room temperature.

## 1. Introduction

High-temperature pressure sensors are of great significance for the monitoring of the dynamic pressures of well logging instruments, chemical reaction kettles, and even combustion chambers. Conventional piezoresistive pressure sensors using silicon diaphragms and piezoelectric pressure sensors are usually unable to withstand temperatures higher than 700 °C [1,2]. Silicon carbide (SiC) is considered a promising material for the fabrication of high-temperature pressure sensors, owing to its mechanical robustness and chemical inertness at elevated temperatures. In addition, the electrical characteristics of SiC feature a wide bandgap, a high-breakdown electric field, and a low leakage current, making it a better candidate than silicon for high-temperature electronic applications [3,4].

High-temperature pressure sensors using SiC-based piezoresistive and capacitive detection mechanisms are available in the 350–600 °C temperature range [5,6,7,8,9]. The silicon substrate and electrical wirings limit their sensing performances at elevated temperatures. Moreover, most of them are sensitive to temperature variations and electromagnetic interference (EMI). Compared with piezoresistive and capacitive sensors, fiber-optic pressure sensors offer high temperature capability, EMI immunity, and corrosion and oxidation resistance [10,11]. Diaphragm-based extrinsic Fabry-Perot interferometer (EFPI) pressure sensors are widely reported for healthcare, automotive, and aerospace applications, in which the diaphragm materials used include polymers [12,13], metals [14,15], silica [16,17], silicon [18,19], and other ceramics [20]. The working temperature of an EFPI pressure sensor is limited by the material of its diaphragm. Pulliam et al. [21] proposed an all-SiC EFPI pressure sensor with a deposited SiC diaphragm and a single-crystal SiC substrate. The SiC pressure sensor shows a nonlinear response that is attributed to the internal stress left during the deposition process.

Several technological challenges remain for the development of all-SiC pressure sensors, such as micromachining for SiC diaphragms and hermetic bonding for sensor cavities. In this paper, we propose an EFPI pressure sensor using a single-crystal SiC diaphragm, which is advantageous for its low internal stress and high mechanical reliability. Both the thin SiC diaphragm and the substrate are fabricated by an ultrasonic vibration mill-grinding method. The sensor cavity is formed by a nickel diffusion bonding technique.

## 2. Design and Calculations

**Figure 1:**
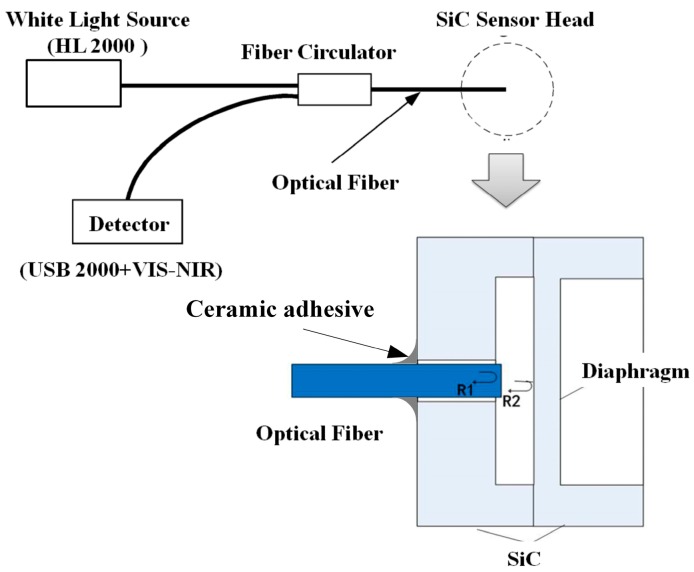
Schematic illustration of the silicon carbide (SiC) sensor head and the measurement scheme.

The EFPI pressure sensor comprises of a SiC diaphragm, a SiC substrate, and an optical fiber as shown in Figure 1. As the diaphragm and substrate of the sensor head are both single-crystal SiC with a same coefficient of thermal expansion, the signal fluctuation induced by a temperature change can be minimized. The cavity length of the SiC pressure sensor varies with the pressure difference between the inner cavity and the outer environment, which can be measured using a scheme shown in Figure 1. The light injected from a white-light source is reflected partly from the fiber endface and the inner surface of the SiC diaphragm. The two reflections then propagate back through the same fiber and generate interference fringes. The reflected lights propagate through a fiber circulator and are detected by a mini-spectrometer. The Fabry-Perot (F-P) cavity length can be determined by demodulating the generated interference fringes [22].

**Figure 2:**
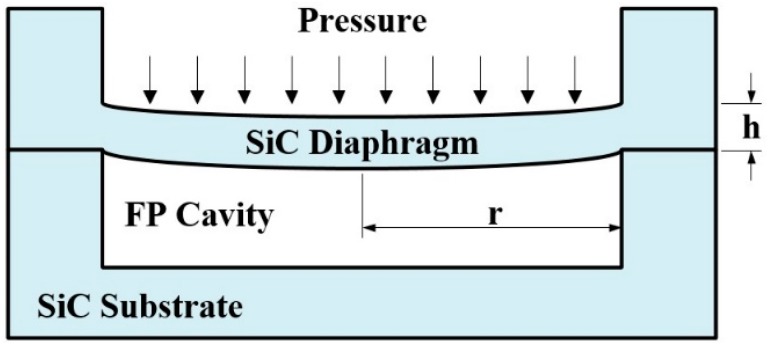
Deflection of a rigidly clamped flat diaphragm under uniform pressure.

The SiC diaphragm is bonded with a SiC substrate as shown in Figure 2. Under uniformly distributed applied pressure, the center deflection of a circular sensor diaphragm is given by the following equation:

(1) $$y_{0}=\frac{3(1-\mu^{2})p}{16Eh^{3}}\times r^{4},$$

where *y*_0_ is the deflection, *p* is the pressure applied on the diaphragm, *μ* is the Poisson’s ratio, *E* is Young’s modulus, and *h* and *r* are the thickness and the effective radius of the diaphragm, respectively. Therefore, the pressure sensitivity (*S*), which is defined as the ratio of the deflection to the pressure difference, can be calculated using Equation (2):

(2) $$S=\frac{y_{0}}{p}=\frac{3(1-\mu^{2})}{16Eh^{3}}\times r^{4}.$$

**Figure 3:**
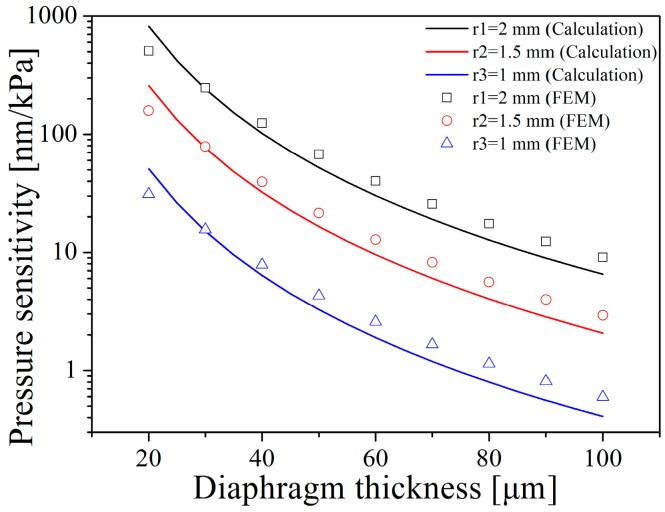
The pressure sensitivity varies with the dimensions of the sensor diaphragm. The dependency is evaluated here by theoretically calculation and by FEM, respectively.

The pressure sensitivity variation with the dimensions of the sensor diaphragm is illustrated in Figure 3, which compares the analytical results obtained from Equation (2) and the finite element method (FEM). In both the calculation and the FEM analysis, the Young’s modulus and the Poisson’s ratio of 6H–SiC are set to 450 GPa and 0.142, respectively. As shown in Figure 3, the pressure sensitivity increases by enlarging the diaphragm radius and decreasing the diaphragm thickness. Considering sensor miniaturization objectives and actual fabrication capabilities, the radius of the diaphragm is set to be 1.5 mm. The displacement resolution of the fiber-optic detection system being approximately 20 nm, the sensor diaphragm should be thinner than 50 µm in order to achieve a detection resolution with a full range of 0–1 MPa. The dimensions of the diaphragm can be further miniaturized with the improvement in micro-grinding techniques. In this case, the thickness of the SiC diaphragm should decrease along with the radius of the sensor diaphragm to sustain the pressure sensitivity.

The frequency response of the SiC diaphragm is of great importance when the dynamic range of the pressure sensor is considered. The diaphragm is defined as a free vibrating circular plate clamped at the edges. Its natural frequency *f_n_* is expressed by Equation (3) [23]:

(3) $$f_{n}=\frac{\alpha h}{4\pi r^{2}}{\left[\frac{E}{3\rho (1-\mu^{2})}\right]}^{1/2}.$$

Here, *ρ* is the density of the SiC diaphragm, and *α* is a constant related to the vibrating modes of the diaphragm and is taken as 10.21 for its fundamental resonance frequency. For a thickness of 50 μm and radius of 1.5 mm, the fundamental resonance frequency of the SiC diaphragm is calculated to be 124.5 kHz, which satisfies the requirements for most of the high-temperature applications.

## 3. Fabrication

**Figure 4:**
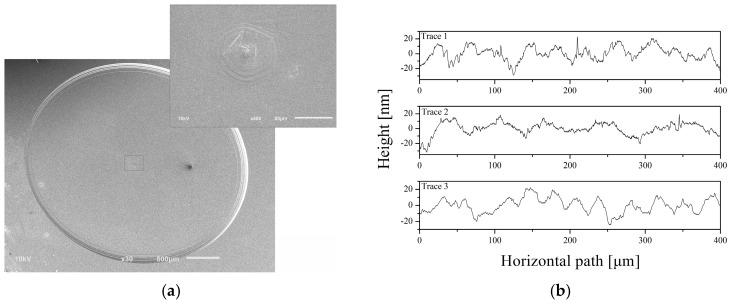
A sensor diaphragm fabricated by the UVMG method with a thickness of 43 μm. (**a**) SEM images; (**b**) measured surface roughness with a trace length of 400 μm.

As single-crystal SiC is a hard and brittle material, the micromachining of the SiC substrate remains a great technological challenge, especially for a thin diaphragm with a low roughness. The ultrasonic vibration machining method has been proposed as an effective cutting process to reduce cutting force and improve surface quality. Ultrasonic vibration mill-grinding (UVMG) can attain a small grinding force and a high machining quality, which plays an important role in hard and brittle material processing [23]. The material removal process has both brittle broken and plastic removal characteristics in the ultrasonic vibration mill-grinding processing of SiC. Grinding and ultrasonic parameters both influence the surface finish in UVMG. The grinding force decreases as the spindle speed increases and the feed rate decreases [24]. As shown in Table 1, with a spindle speed of 45,000 rpm, the surface roughness decreases by applying an ultrasonic vibration amplitude of 0.5 μm. In addition, a lower surface roughness can be obtained with a smaller axial feed rate. A sensor diaphragm with a thickness of 43 μm is illustrated in Figure 4a. The inset in Figure 4a shows a small surface lay in the center of the diaphragm, which can be removed by modifying the computer numerical control (CNC) programs. Figure 4b shows the profile of a machined surface measured by a surface profiler (D-600, KLA-Tencor, Milpitas, CA, USA), and the roughness (Ra) is as low as 19 nm.

**Figure 5:**
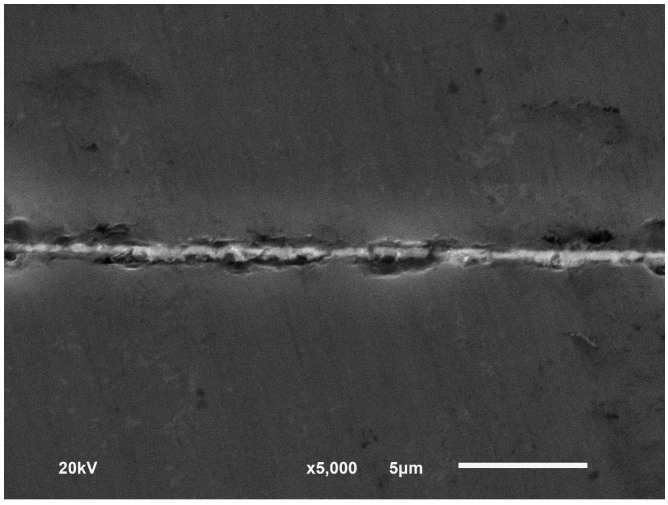
Bonding interface of the SiC layers using the nickel diffusion method with a temperature of 900 °C and a pressure of 42 MPa.

The sensor cavity with a radius of 1.5 mm was fabricated on a SiC substrate using the UVMG method. A 150-μm-diameter through hole was formed in the SiC substrate using a picosecond pulsed laser machining technique to fit the optical fiber. In order to achieve a sealed reference cavity, it was necessary to make a hermetic bonding of the SiC layers and mount the optical fiber with a high-temperature ceramic adhesive. The nickel diffusion bonding method [25] was used to bond the SiC diaphragm and the substrate together. Both of the SiC layers were sputtered with a 500-nm-thick nickel film, and then bonded at a high temperature of 900 °C with a pressure of 42 MPa for 30 min. A cross-sectional view of the bonded interface is shown in Figure 5. 

**Figure 6:**
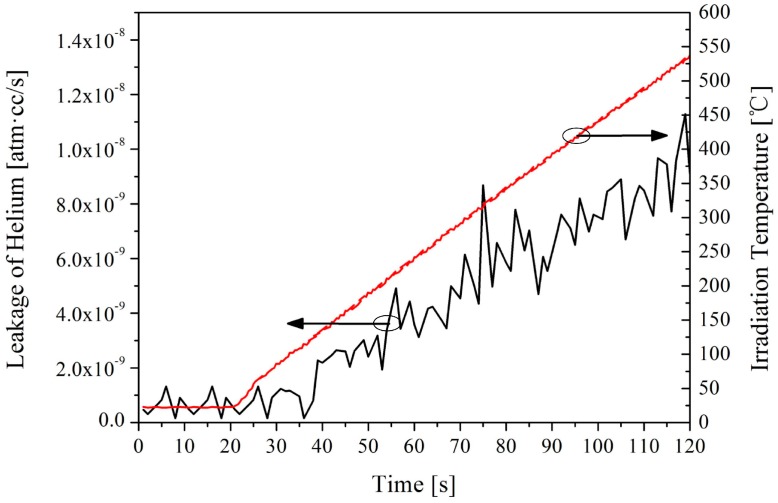
Leakage rate of the sensor cavity measured by helium detector variation with the ambient temperature.

In order to verify the sealing performance of the Ni diffusion bonding, a SiC diaphragm and a SiC substrate were fabricated by UVMG and bonded to form a sensor cavity, in which no through hole is formed. The leakage rate variation in the ambient temperature was measured using a helium detector. As shown in Figure 6, the leakage rate of the sensor cavity increases abruptly at 127 °C and reaches 1.1 × 10^−8^ atm·cc/s at 540 °C. This suggests that the bonding conditions should be improved to achieve a low leakage to make the sensor functional at higher temperature conditions.

**Figure 7:**
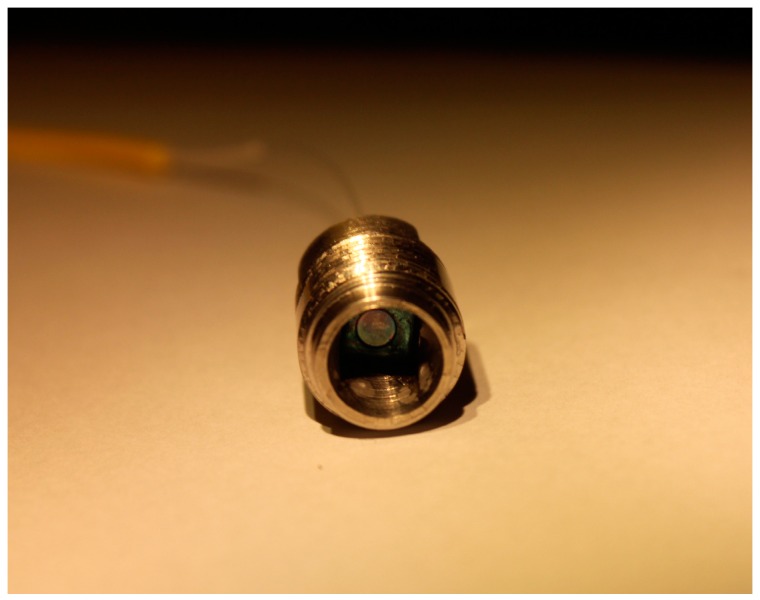
The fiber-optic pressure sensor with a full SiC sensor head.

The SiC pressure sensor head is of square shape with a side length of 6 mm, and a thickness of approximately 660 μm. Considering the coefficient of thermal expansion, the sensor head is then assembled into a molybdenum package fitting with M14 threads as shown in Figure 7. Finally, a single mode optical fiber is assembled into the sensor head using a micro displacement worktable and sealed with a high-temperature ceramic adhesive to achieve the prototype device.

## 4. Characterization and Discussions

**Figure 8:**
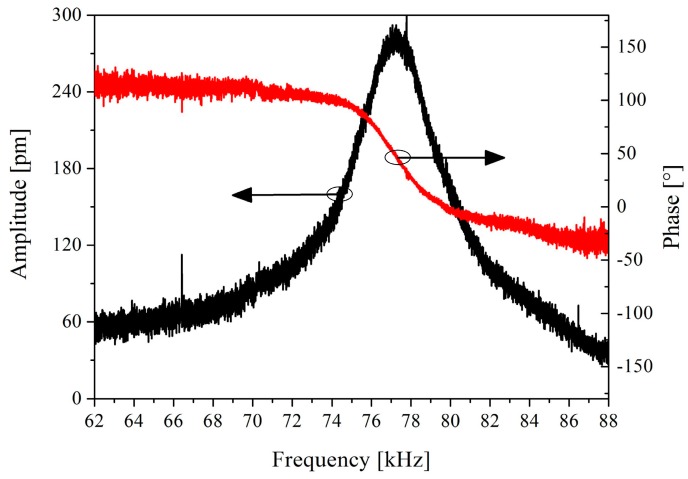
Frequency response of the SiC diaphragm with a thickness of 43 μm.

The mechanical frequency response of the sensor diaphragm was measured using a laser Doppler micro system analyzer (MSA-500, Polytec, Waldbronn, Germany). As shown in Figure 8, the fundamental resonance frequency is approximately 78 kHz. The resonance frequency of the diaphragm is sufficiently high, as the pressure measurement for many high-temperature chambers is in the 0–10 kHz dynamic range.

**Figure 9:**
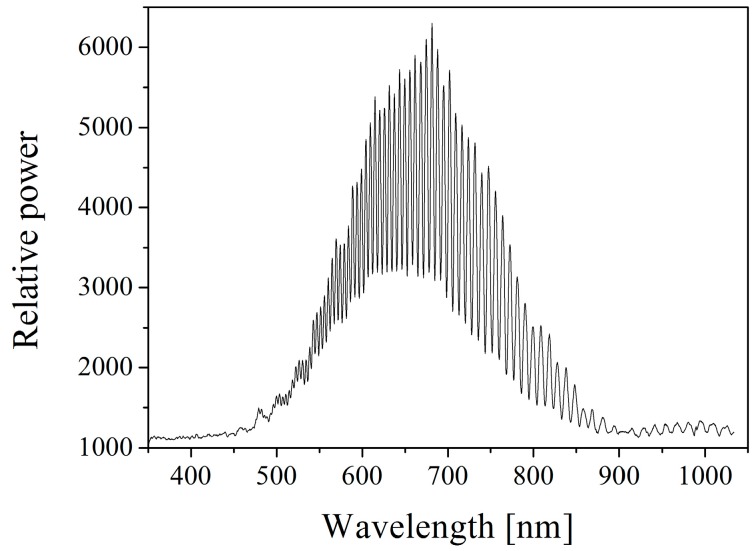
A standard interference spectrum of the EFPI pressure sensor with a cavity length of approximately 35 μm.

**Figure 10:**
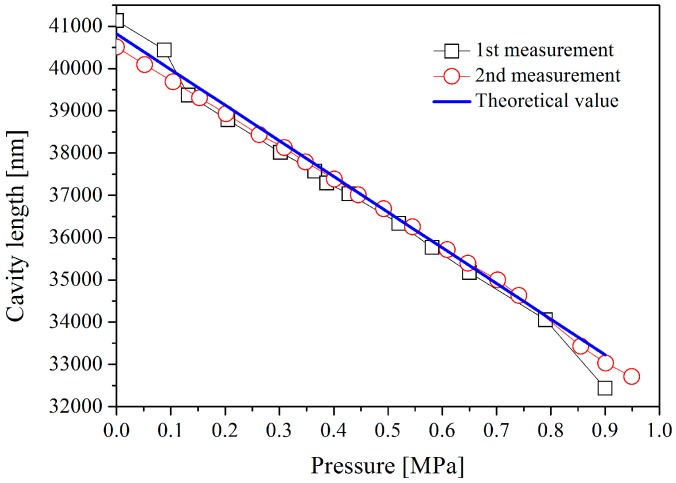
Cavity length as a function of pressures measured at room temperature.

**Figure 11:**
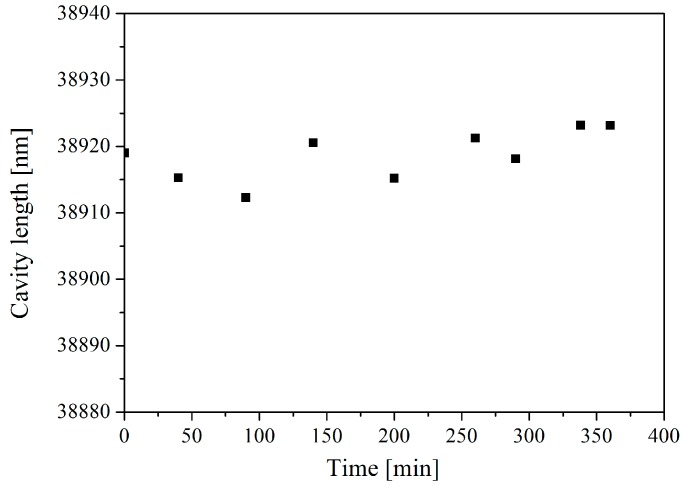
The fluctuation of the cavity length while sustaining the pressure at 0.2 MPa for 400 min at room temperature.

The sensor was connected to an adjustable pressure chamber for calibration. A continuous white light source, a circulator, and a spectrum analyzer were used for cavity length detection using the scheme shown in Figure 1. A standard interference spectrum of the EFPI pressure sensor is illustrated in Figure 9, which shows obvious interferences, with an F-P cavity length of approximately 35 μm. As shown in Figure 10, the cavity length decreases linearly with the increase of the applied pressure from 0.1 MPa to 0.9 MPa. The fluctuation of the cavity length is approximately 0.27% F.S. (full scale) as shown in Figure 11, while the pressure is held at 0.2 MPa for 400 min at room temperature. 

A conventional single-mode SiO_2_ optical fiber was used for this prototype device. In order to explore the temperature limit of the pressure sensor, a sapphire-based optical fiber should be utilized to replace the SiO_2_ fiber. As a result, the characterization of this prototype device was only conducted at room temperature. The fiber-optic SiC pressure sensor should be calibrated at high temperature conditions in future works.

## 5. Conclusions

An all-SiC structure diaphragm-based EFPI pressure was developed. The SiC diaphragm was fabricated with a thickness of 43 μm and a surface roughness of 19 nm using ultrasonic vibration mill-grinding. The sensor head was formed using a nickel diffusion bonding technique. The pressure sensor shows a good linearity in the range of 0.1–0.9 MPa, with a resolution of 0.27% F.S. at room temperature. As the all-SiC pressure sensor needs to work at temperatures over 1000 °C, future studies must work to improve the bonding process and investigate the high temperature characteristics of the pressure sensor.

## Figures and Tables

**Table 1:** Variations of sensor diaphragm roughness with the feed rate and amplitude of ultrasonic vibration, in the case that the spindle rotation speed is 45,000 rpm.

No.	Wheel Mesh Size	Spindle Speed (rpm)	Axial Feed Rate (μm/s)	Vibration Amplitude (μm)	Roughness Ra (nm)
4	600#	45,000	0.16	0	31
5	600#	45,000	0.32	0	76
6	600#	45,000	0.64	0	192
7	600#	45,000	0.16	0.5	19
8	600#	45,000	0.32	0.5	25
9	600#	45,000	0.64	0.5	31
